# Supplementary figures and images for: Effect of habitat fragmentation on rural house invasion by sylvatic triatomines: A multiple landscape-scale approach
Source: PLoS Negl Trop Dis. 2021 Jul 14;15(7):e0009579. doi: 10.1371/journal.pntd.0009579 (PMC8312942; doi:10.1371/journal.pntd.0009579)

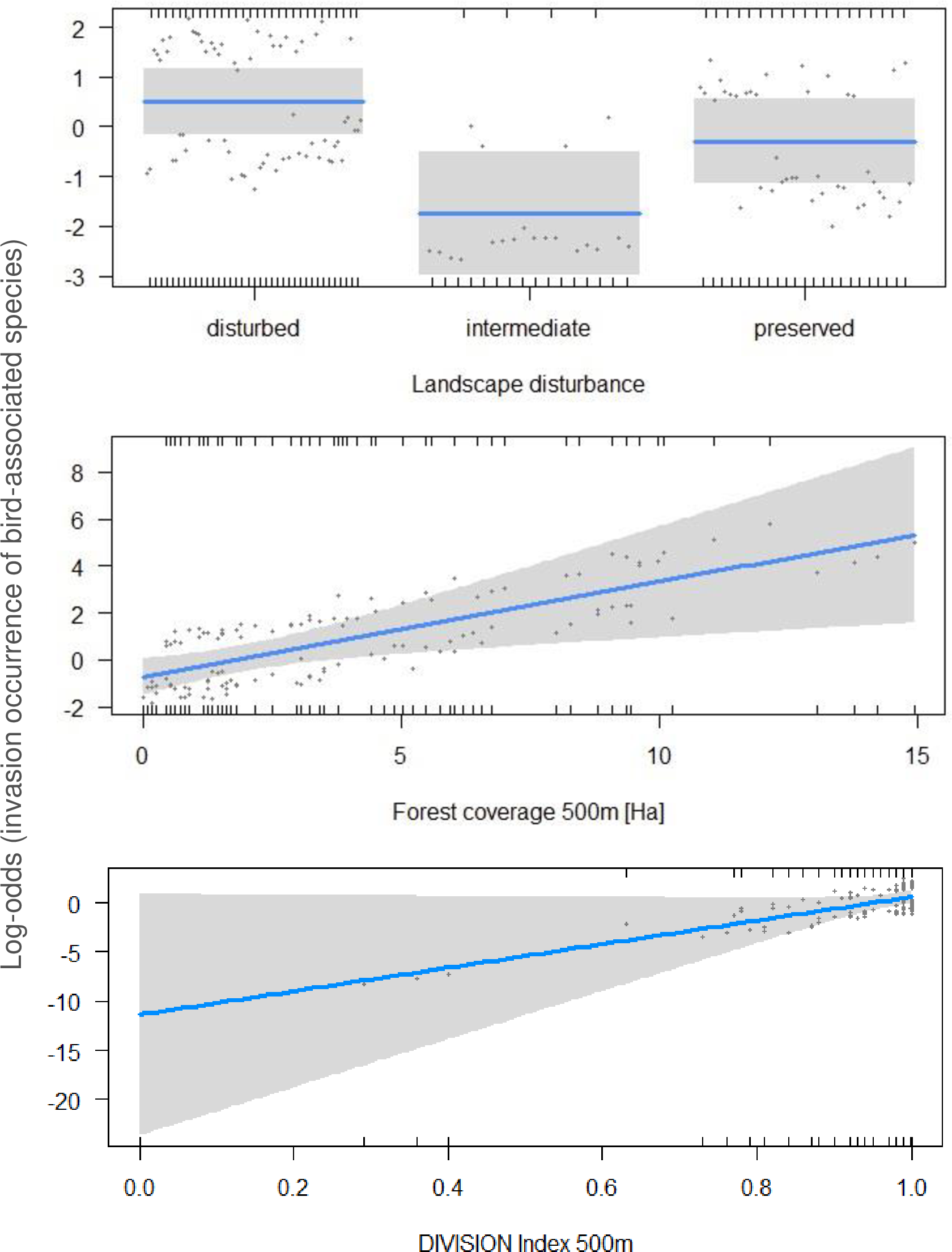

Supplement: S1 Fig — The blue line corresponds to the predicted values, the gray band its 95% confidence intervals, the gray points are the partial residuals, and the upper and lower lines are the observed values of the response variable. (TIF) [file pntd.0009579.s002.tif]

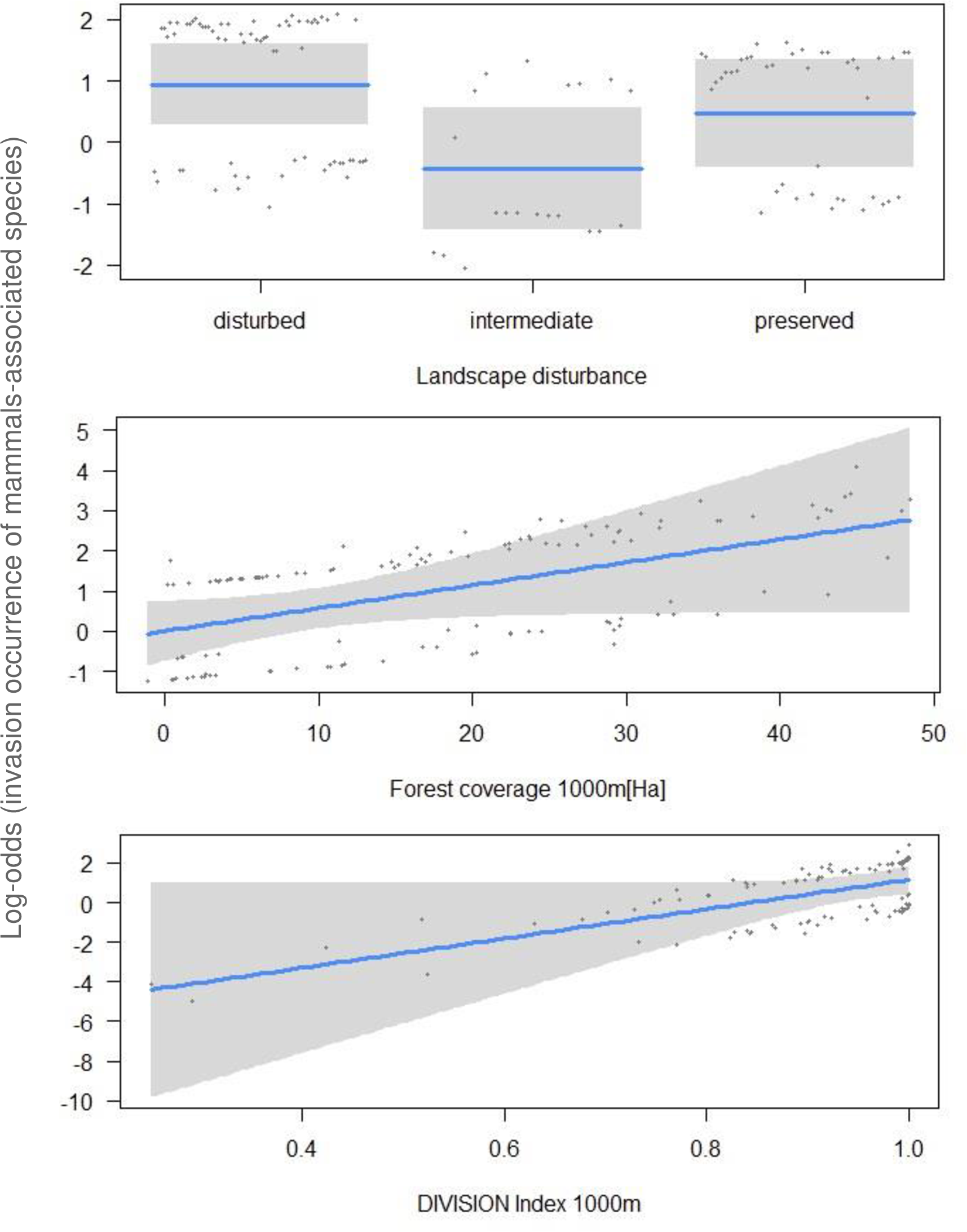

Supplement: S2 Fig — The blue line corresponds to the predicted values, the gray band its 95% confidence intervals, the gray points are the partial residuals, and the upper and lower lines are the observed values of the response variable. (TIF) [file pntd.0009579.s003.tif]

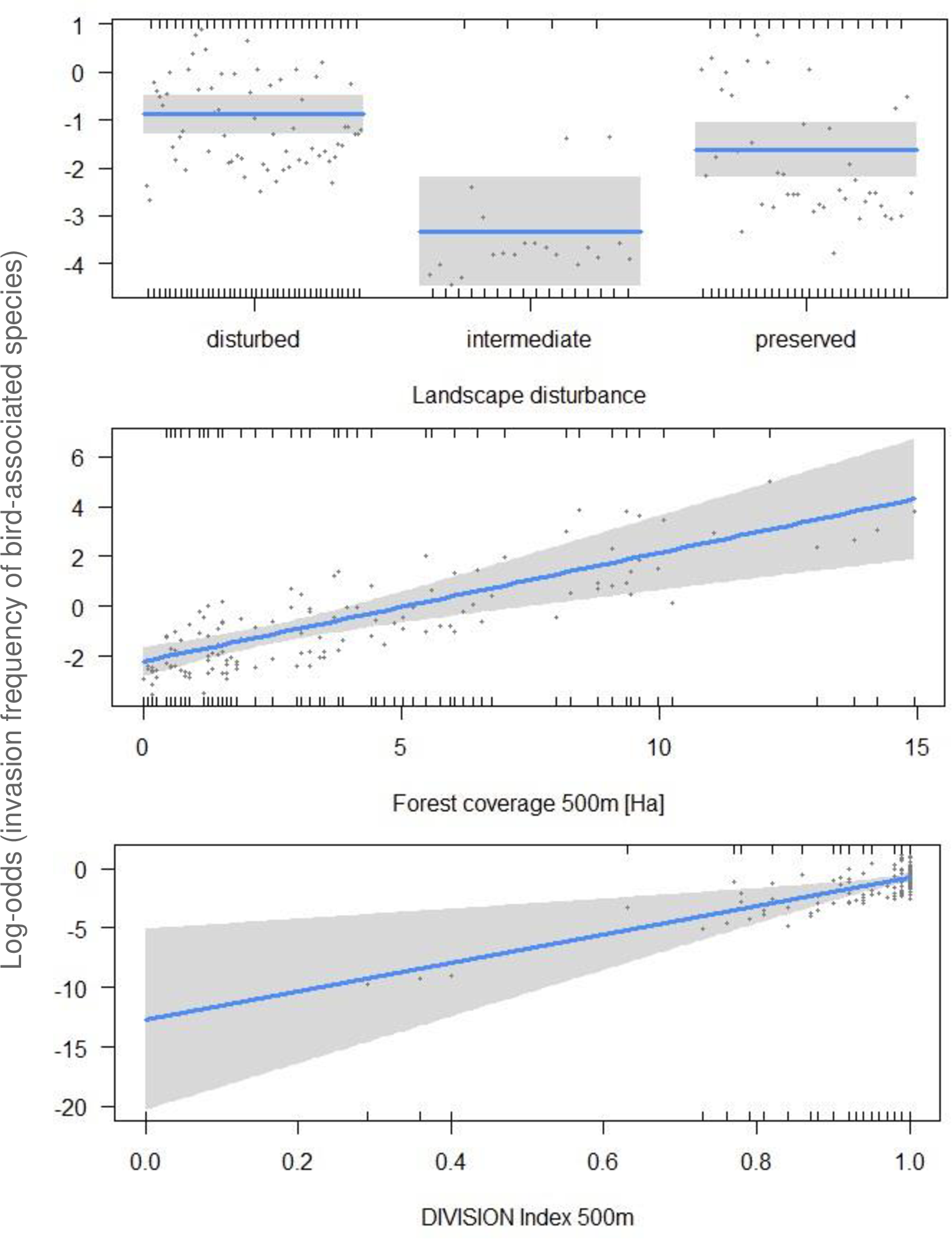

Supplement: S3 Fig — The blue line corresponds to the predicted values, the gray band its 95% confidence intervals, the gray points are the partial residuals, and the upper and lower lines are the observed values of the response variable. (TIF) [file pntd.0009579.s004.tif]

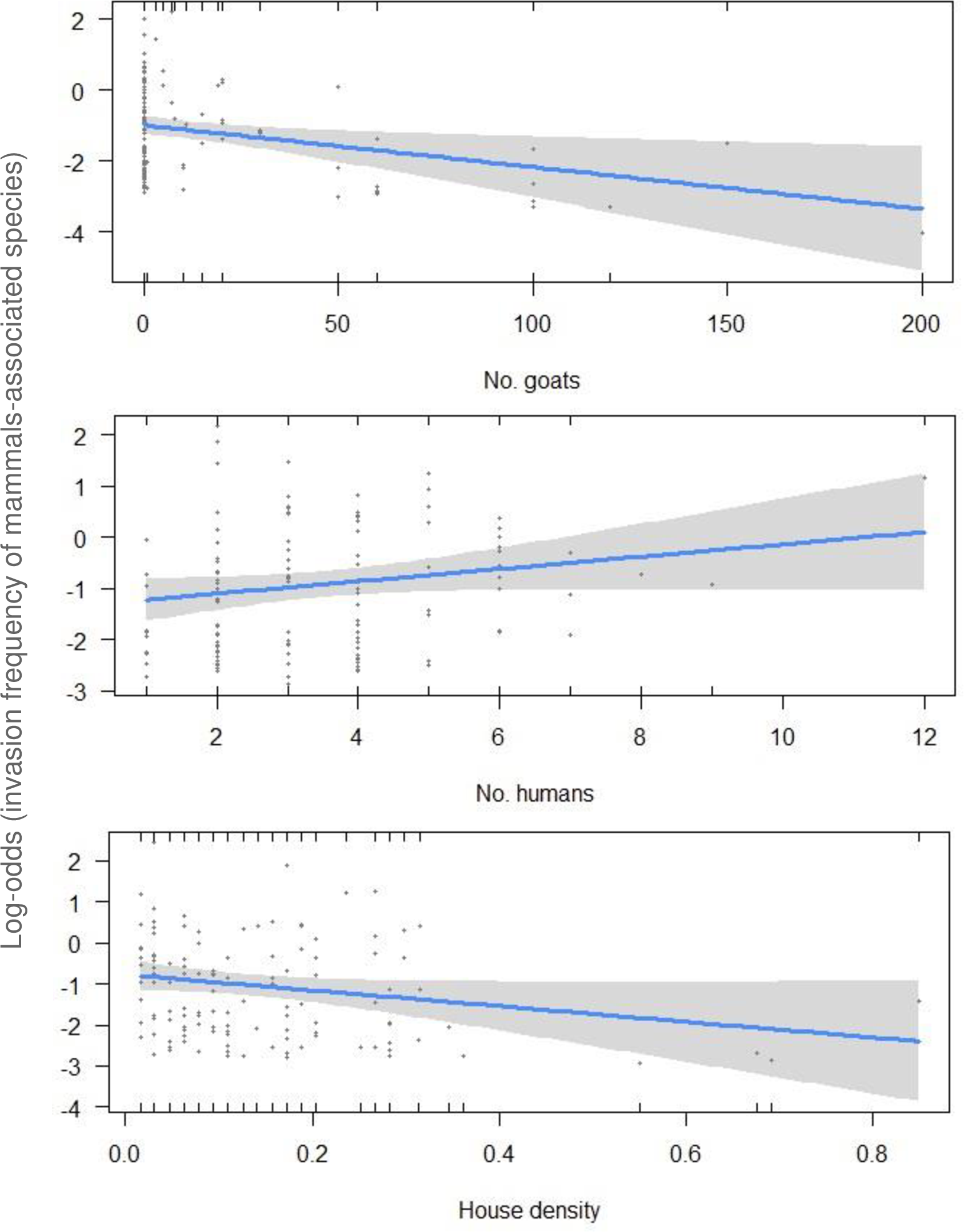

Supplement: S4 Fig — The blue line corresponds to the predicted values, the gray band its 95% confidence intervals, the gray points are the partial residuals, and the upper and lower lines are the observed values of the response variable. (TIF) [file pntd.0009579.s005.tif]
