# Supplementary material for: Effect of habitat fragmentation on rural house invasion by sylvatic triatomines: A multiple landscape-scale approach
Source: PLoS Negl Trop Dis. 2021 Jul 14;15(7):e0009579. doi: 10.1371/journal.pntd.0009579 (PMC8312942; doi:10.1371/journal.pntd.0009579)
Supplement: Attachment — Submitted filename: Response to reviewers.docx [file pntd.0009579.s006.docx]

Dear reviewers,

Thank you very much for the comments and suggestions that will certainly clarify the understanding of the manuscript.

Regarding the comments of reviewer # 1,

*R#1:The results presented are interesting and adequately match the analyses plans. Results are also clear and well presented in different Tables, and adequately discussed. Perhaps Fig 2 may need to be improved for better visual clarity.”*

Thanks for the suggestion. The resolution of Figure 2 was improved for better visual clarity and submitted in TIFF format with a resolution of 300 dpi, which is the minimum resolution according to the instruction of the journal.

Regarding the comments of reviewer # 2,

*R#2:The title of the manuscript is not fully reflecting it contain. The authors indicated that the manuscript report a modeling experiments, however the text did not put emphasize of modelling studies either in the introduction or in the discussion. No comparison with existing models was conducted. I will suggest to authors to replace the word “modeling” in the title by “Enhancing the understanding”. The methodology is about study sites, and data analysis and not modelling experiments and the whole line 547-552 should be remove from the text.”*

Thanks for the suggestion. The title was modified as follows: “Habitat fragmentation effect on rural house invasion by sylvatic triatomines: a multiple landscape-scale approach”; and the whole line 547 – 552 was removed from the discussion section.

*R#2:”The text further lack justification why the selected methods were used for the analysis. Why the maximum likelihood method for classification was used instead of more robust technique such as random forest? Please justify the choice of the methodology”*

Thanks for the comment. We decided to use maximum likelihood for comparability reason. Thematic maps obtained with the method were used in previous spatial studies related to the ecology of Chagas disease vectors in the region (Moreno et al. 2010; Porcasi et al. 2011; Weinberg et al. 2018) as well as in the latest update of floristic composition of the major native woody vegetation types in the southern Great Chaco and Espinal (Hoyos et al. 2013; Cabido et al. 2018). In these studies, the Maximum Likelihood routine, using training sites for validation, was used. In addition, this classification method has been used in recent studies to characterize the association between environment variables and other vectors such as mosquitoes (Vergara Cid et al. 2013; Estallo et al. 2018; Benitez et al. 2019). Although machine learning classifiers like random forest are more robust than other classifiers, some authors states that very similar results can be obtained from both approaches when a non-complex scene is used (Palm and Mather, 2003). Therefore, taking into account the type of coverage classes selected for the thematic map (that does not require a level of detail to identify tree species or types of crops) and considering all the previous studies mentioned before, the Maximum Likelihood classification method was selected.

Regarding the comments of reviewer # 3,

*R#3:” There are some grammar errors in English (e.g. lots of subject-verb agreement errors)- I am sure I only caught a few and not all of these errors so this should be double chcked by English editors.”*

*line 131- change 'refuges' to 'refuge'*

done
*line 128- 'increases' change to 'increase'*

done

*line 591- put a comma after 'besides'*

done

Thanks for the suggestions. English grammar was revised and some parts of the text were rephrased after the comments by reviewers #2 and #3. All modifications are visible through the use of the track changes tool.

*R#3:”line 168- only looking for T. cruzi by microscopic evaluation of bug feces can markedly underestimate infection rates , multiple studies confirm this. you may want to not present this microscopic data without molecular confirmation. The T. cruzi results tested microscopically add nothing to this study so I suggest that they don't include this (only microscopic analysis was done on some bugs and actually molecular analysis is at this point the 'norm' as there are too many false negatives with microscopic analysis).”*

Thanks for the suggestion. We decided not to present the results of T. *cruzi* infectivity using only microscopic evaluation. This change does not affect the main results and conclusions of the study.

*R#3: For table 2- can you please show the totals across each landscape (adding up the communities for each habitat type?)- it would be helpful to know the mean number of goats, chickens, dogs, etc for each household not just the total across all households.”*

Table 2 was modified including the totals across each landscape disturbance category and the mean number of domestic hosts per house.

*R#3: In the methods, can you describe a bit more what a 'typical' house might look like (construction, lights, windows, permeability to invasion of bugs?) Do all households have electricity?”*

In line 143-151 we added the description of the “typical” rural house of the area: “*Most of the traditional rural houses in the study area (historically built with adobe walls and local vegetation on the roofs) were replaced, in the last decade, by dwellings with brick plastered walls and galvanized aluminum or cement roof. Although all rural houses visited during field work had electricity, the use of mosquito nets on doors and windows is very rare.”*

*R#3: Minor comments
line 146- can you describe the patterns of habitat loss in Cordoba province?*

In lines 158-162 we described the pattern of habitat loss in the northern region of the Province of Córdoba: “*Approximately 85 % of the originally undisturbed Chaco forest of the northern of Córdoba province has been transformed into a highly fragmented mosaic of isolated patches of forest, dense thorny shrubs, seminatural grassland and a large surface of cultivated land. These modifications varied in intensity, with an east-west gradient associated with the rainfall regime. [33].”*

*line 164- why were these seasons chosen for field sampling?*
The justification for the selection of the warm season for field sampling was added in lines 182 – 184: “*Field sampling was conducted during the warm season (November - March), because active dispersion of triatomines in the Chaco region is more frequent during the warmer months of the year.”*

Additionally, this work is part of a much broader project aiming to understand and characterize the dispersal capacity of triatomines species in different environments both at the beginning and at the end of the warm season.

*R#3: Some of the figures (graphs) appear a bit pixellated- but that could be the quality of my computer- would require prof. editorial check at PlosNTDS*

All the figures submitted are in accordance with the specifications of the figures described in the instruction of the journal (TIFF format, 2250 x 2625 pixels and 300 ppp).

We deeply appreciate your reviews and comments, they helped us improve the manuscript.

With kind regards,

Biol. Cardozo Miriam

Biol. Fiad Federico

Dra. Crocco Liliana B

Dr. Gorla David E.
